# Prediction of individual, community and societal resilience in the Czech Republic compared to Slovakia during the war in Ukraine

**DOI:** 10.1186/s12889-024-18075-y

**Published:** 2024-02-23

**Authors:** Alice Koubová, Shaul Kimhi

**Affiliations:** 1 Institute of Philosophy , Czech Academy of Sciences, Prague, Czech Republic; 2 Sackler Faculty of Medicine, Israel School of Public Health, Tel Aviv University, Tel Aviv, Israel

**Keywords:** War in Ukraine, Individual, Community and societal resilience, Hope, Morale, Well-being, Distress symptoms, Sense of danger, Perceived threats

## Abstract

The present study examines, as research questions, which and to what extent psychological and demographic variables significantly predict individual, community, and societal resilience among a sample of Czech Republic adults (*N* = 1,100) six months after the Russian invasion of Ukraine. The research tools included the following scales: Societal, community, and individual resilience; hope, well-being; morale; distress symptoms; a sense of danger; and perceived threats. The results indicated the following: (a) Correlation analysis shows that resilience is significantly and positively correlated with supporting coping factors and significantly and negatively correlated with suppressing coping factors. (b) A comparison of supporting coping indicators (hope, well-being, and morale) and suppressing coping indicators (distress symptoms, sense of danger, and perceived threats) in the Czech Republic with those variables in Slovakia and Israel indicated that Israel reported higher resilience, higher supporting coping indicators, and lower suppressing coping factors. Three-path analysis among the Czech sample indicated that the best predictor of SR was the level of hope, the best predictor of CR was morale, and the best predictor of IR was the sense of danger. In an attempt to explain these findings in the discussion section, we refer to the background of Czech society and a possible connection to the findings.

## Introduction and background

On February 24, 2022, the Russian forces invaded Ukraine, thinking that in a short time, they would take over the country and it would become, in practice, a Russian territory. According to several experts, Putin’s invasion appears to have been based on a risk-taking policy [1] and a misguided assumption that Ukrainians would surrender quickly and without protesting– either because of fear or because of the wish to welcome Russian soldiers as liberators– and that the “special operation” would last a short time [2]. However, the war has continued for more than a year, and the end is as yet not in sight.

This war affected the populations of Ukraine and European countries, as well as the global community at large, in varied aspects of life. Among these aspects, it is possible to mention the food security of countries [3], the rise of energy prices [4], economic prices [5], problems regarding physical health [6], an unprecedented migration crisis [7], and other war’s impacts and aspects [8–12].

Against this background of a war in Ukraine, the present study aims to examine individual, community, and societal resilience in the Czech Republic. Specifically, the study aims to examine to what extent psychologically supporting coping indicators (hope, well-being, and morale) and suppressing coping indicators (distress symptoms, sense of danger, and perception of threats), which were used in previous studies as distinct predictors of resilience, will play a similar role in the Czech Republic. In addition, we will examine to what extent acceptable demographic variables will predict resilience.

Previous studies examined resilience following the COVID-19 pandemic [13]; [14], military conflicts and terror [15], and the climate crisis [16], comparing coping with terror and COVID-19 [17], as well as the war in Ukraine [14]. Based on these studies ( [13–17]), it is possible to suggest some conclusions: (a) There are considerable interpersonal differences in the level of resilience of humans, as well as between different countries and/or cultures. (b) Resilience tends to decrease in times of crisis. It is usually a slow and continuous process. (c) Women tend to report higher levels of distress than men, but not necessarily lower resilience. (d) The age group reporting the highest resilience is the older age group, while those aged 30–40 reported the lowest level of resilience.

### Resilience

The concept of resilience has received much attention in the professional literature in recent years. The reasons for this are related to a series of global threats, such as the climate crisis, the COVID-19 pandemic, and wars such as the war in Ukraine. In other words, the many serious threats to the world population might explain the “popularity” of the resilience concept. However, resilience has many definitions, and researchers from different fields define it to different extents [18]; [19]; [20]. In the social sciences, it is possible to find a variety of definitions that refer to the ability of humans (individuals, communities, and entire societies) to cope with adversities (human-made or natural-made) and threats with maximum success and to recover or return to daily life as fast as possible after the removal of the threat [21]. A review of resilience studies indicates that it is acceptable to refer to three types of resilience: individual, community, and societal (national) resilience [17]:

#### Individual resilience (IR)

This refers to the individual’s ability to cope with various adversities and to recover at the end of the event(s) [22]. Individual resilience is a central component in an individual’s ability to cope with various adversities. However, studies show that the correlations between individual, community, and societal resilience are significant but not very high, and it seems that these are independent structures [23].

#### Community resilience (CR)

Community resilience refers to a community that lives in a certain geographic area and has an authority that is an address for various emergencies. In the professional literature, there are many definitions of community resilience [24]. In general, CR refers to the ability of a community, as a social body, to cope with adversities and recover in the shortest time possible [20]; [25].

#### Societal resilience (SR, former national resilience)

SR refers to the ‘perceived ability of the society to successfully deal with adversities and quickly recover after the threat has been removed’ [15] (p. 2). Earlier studies indicated that SR contains four factors: trust in the country’s leaders, trust in state institutions, social unity, and willingness to contribute to the country or patriotism [26].

### Supporting and suppressing coping indicators

Factors supporting coping If the higher the factors are, the more they indicate better coping. In the current study, we examined three factors: hope, well-being, and morale. Factors inhibiting coping are factors that the higher they are, the more they indicate less successful coping. In the current study, we examined three: distress symptoms, a sense of danger, and perceived threats.

#### Hope

Earlier studies and theoretical approaches refer to hope as an important way to cope with distress or adversity: to expect some positive result, sometimes in the future [27]; [28]. According to researchers, coping and hope are mutually dependent, as hope underlies all coping efforts. Previous studies have indicated the great importance of hope in dealing with various adversities [29].

#### Well-being

According to Levaot [30], emotional well-being includes a positive balance of pleasant to unpleasant affect and a cognitive appraisal of satisfaction with life in general. Well-being is a general indicator of the quality of life, according to a person’s evaluation. Well-being is a good indicator of a person’s quality of life [31], as well as resilience [13].

#### Morale

Morale is regarded by Weakliem and Frenkel [32] as a general term for positive feelings about the prescribed activities of the group. Webster’s Dictionary defines morale as “the mental and emotional condition (as of enthusiasm, confidence, or loyalty) of an individual or group concerning the function or tasks at hand.” Positive morale is usually characterized by discipline, confidence, and willingness to perform [33]. According to Din and Khuwaja [34], high morale refers to the adjectives happy, confident, and appreciated, whereas sad, depressed, and unrecognized adjectives are related to low morale. A higher level of morale is likely to be associated with a more positive future orientation and with better resilience in hard times.

#### Distress symptoms

Symptoms of anxiety and depression deal with reactions among people, in most cultures, when coping with adversities of all kinds (natural and human-made disasters), and they signify the difficulty of humans to cope with these adversities [30]; [35].

#### Sense of danger

Sense of danger refers to the extent to which the individual perceives his world as being at risk. Studies have indicated that a low sense of danger and few worries about potential threats are positively related to resilience and the ability to recover post-traumatically and negatively related to stress symptoms: the higher the feelings of danger, the lower the ability to recover and the more stress symptoms [36].

#### Perceived threats

Threats can be regarded as dangers perceived by the individual as validating him and/or those close to him [16]. The dangers can be of different types, from existential dangers (war, terrorism, natural disasters) to threats to his financial situation, social status, or good name [30]. The current study focuses on examining the effects of the Ukrainian War on the civilian population in the Czech Republic. Perceived threats were examined in this study as a negative indicator of resilience and successful coping.

The purpose of the study is to examine the associations between resilience and coping measures, in response to an imminent war and a situation of uncertainty. For a better understanding of the meaning of the results, as well as to obtain a proportion of the findings, we compared the results of the Czech Republic with the results of two other countries: Slovakia and Israel.

## Method

### Sample and sampling

Six months after the invasion of Russia into Ukraine, we sent a uniform questionnaire to a sample of adults in the Czech Republic population (*N* = 1,011). The data were collected via an internet panel company. Recent studies have shown that the results from a sample based on an internet panel are not fundamentally different from a sample that is based on other methods (e.g., Bach et al., 2023). The panel Internet company possessed a database of tens of thousands of individuals from the respective societies. The current sample included residents from all common demographic and socioeconomic sectors, such as age, gender, education, family income, family status, and level of religiosity. The internet panel randomly samples the respondents from the extensive panel, following the established rules such as the equal representation of gender and the number of subjects (see Table 1). The research questionnaire (including all the scales) was approved by the university ethics committee (approval no. 005146-1).

**Table 1:** The Czech Republic’s socioeconomic and demographic characteristics (*N* = 1,011)

Scale	Details	n	%	M	S.D.
Age group^1^	18–30	167	16.5	49.22	15.98
	31–40	157	15.5		
	41–50	206	20.4		
	51–60	169	16.7		
	61–70	222	22		
	71 - above	90	8.9		
Gender	1. Female	515	50.9		
	2. Male	496	49.1		
Education	1. Elementary school	61	6.0	2.63	1.21
	2. High school	641	63.4		
	3. More than high, no academic	88	8.7		
	4. Bachelor’s degree	53	5.2		
	5. Master or above	168	16.6		
Family Status	1. Married	487	48.2		
	2. Single	279	27.6		
	3. Divorced/widow	207	20.5		
	4. Other	38	3.8		
Children	1. No	268	26.5	2.46	1.11
	2. One	201	19.9		
	3. Two	387	38.3		
	4. Three	117	11.6		
	5. Four or more	38	3.8		
Family income compared to the average	1. Much below M	96	9.5	2.98	1.11
	2. Lower than M	204	20.2		
	3. Average	365	36.1		
	4. Higher than M	313	31.0		
	5. Much higher above M	33	3.3		
Religiosity	1. Secular	640	63.3	1.46	0.68
	2. Traditional	286	28.3		
	3. Religious	72	7.1		
	4. Very religious	13	1.3		

^1^ The average scale score was divided into four categories.

### Tools

All the scales we used in this study are existing scales that we used in previous studies and were found to be valid in several countries after being translated into the language of the country [15]; [26]. We used the following scales in the current study: (a) three types of resilience scales (societal, community, and individual); (b) three supporting coping indicators (level of hope, well-being, and morale); and (c) three scales of suppressing coping indicators (distress symptoms, sense of danger, and perceived threats). The reference to each of the scales appears after the name of the scale.

#### Societal resilience [37]

Ten items (suh as “The Czech Republic is my home and I do not intend to leave it”) were included, ranging from 1 = strongly disagree to 6 = strongly agree. The Cronbach’s alpha reliability of this scale was α = 0.90.

#### Community resilience [38]

Nine items (such as “I can trust people in my community to come to my aid in case of crisis”) were included, ranging from 1 = do not agree at all to 5 = agree to a very large extent. The Cronbach’s alpha reliability of this scale was α = 0.88.

#### Individual resilience [39]

The two items (such as “I can adapt when changes occur”) suggested by these authors were used, ranging from 0 = do not agree at all to 4 = agree to a very large extent (for the analysis of the data, we have recoded the scale to 1–5). The Cronbach’s alpha reliability of this scale was α =.73.

#### Hope [40, 3, 41]

Three items were adapted in their context to a security threat (for example, “I have hope that I will emerge strengthened from the Ukraine war”), ranging from 1 = very little hope to 5 = very much hope. The Cronbach’s alpha reliability of this scale was α = 0.91.

#### Well-being [42]

In this scale, the subject is asked to answer 5 statements, each of which describes his situation in a different area of life (family, society, leisure life, employment, and so on). The scale extends from 1 = very bad to 6 = very good. This measurement scale was found to have good support in previous studies, and its reliability in the present study was found to be high (α = 0.83).

#### Morale

We used the following item to measure the level of Morale: “What is your Morale (personal mood) these days?” The answer to the question was given on a 5-point Likert scale, ranging from 1 = very bad to 5 = very good.

#### Distress symptoms [1]

Four items relating to anxiety (for example, “I feel such restlessness that it is impossible to sit in one place”) and 4 items relating to depressive symptoms (such as “I feel a lack of interest in my world”) were included, ranked on a 5-point Likert scale, ranging from 1 = not at all to 5 = to a very large extent. The internal reliability, measured by Cronbach’s alpha, of the scale was very good (α = 0.91).

#### Sense of danger [43]; [15]

The scale of the sense of danger index used in the present study includes 5 items and ranges from 1 = not at all to 5 = very much. The scale was used by us in previous studies and was found to be reliable and valid. In the current study, the internal reliability of the scale was found to be very high (α = 0.90).

#### Perceived threats [44]

In the current study, we asked the respondents to rate five threats: economic, social, security, health, and a threat arising from the political situation regarding the war in Ukraine. The answer to each of the questions regarding each of the four threats was given on a 5-point scale, from 1 = not threatening at all to 5 = very threatening. In the current study, the internal reliability of the scale was found to be very high (α = 0.85).

### Demographic characteristics

The socioeconomic characteristics that we measured were level of religiosity (1 = secular, 4 = very religious) and average family income compared with the average income in the Czech Republic (1 = much lower than the average to 5 = much higher than the average). The demographic characteristics were education (1 = elementary, 5 = Master’s degree and above), gender (1 = female, 2 = male), and age (divided into six age groups). These socioeconomic and demographic data are common in social sciences studies [45].

The socioeconomic and demographic data that characterize the Czech sample are presented in Table 1: Wide age sample, a similar percentage of males and females, 21.8% with academic degrees, half of the sample are not married, a similar percentage of the respondents regarding the above, and below family average income, above 60% of the respondents reported that they are secular.

### Statistical procedures

To examine the findings, we used the following procedures: (a) Pearson correlation among the study’s psychological variables. (b) Analysis of variations comparing the Czech Republic’s resilience and coping indicators with those variables in Slovakia and Israel. (c) Three path analyses to examine the prediction of IR, CR, and SR by psychological variables, demographic variables, and a combination of both (see Table 2).

**Table 2:** Standardized estimated path analysis of significant psychological and demographic variables predicting three types of resilience in the Czech Republic

Predictor	IR Estimate	CR Estimate	SR Estimate
*Psychological variables*			
Hope	0.10***	0.25***	0.27***
Well-being	0.19***	0.23***	− 0.01
Morale	0.02	0.44***	0.07*
Distress	− 0.09*	0.04	− 0.09**
Sense of danger	− 0.25***	− 0.06*	0.09*
Threats	0.04	− 0.11***	− 0.32***
% variability	15%	22%	26%
*Socioeconomic and demographic characteristics*			
Religiosity	− 0.13***	0.11***	0.07*
Family income	0.16***	0.14***	0.13***
Education	.11**	0.03	0.01
Gender	0.06	0.02	− 0.07*
Age	0.16***	0.06	0.05
% variability	8%	3%	2%
*Psychological and Demographic*			
Hope	0.11***	0.24***	0.28***
Well-being	0.18***	0.22***	− 0.02
Morale	− 0.01	0.14***	0.07*
Distress	−.09*	0.04	− 0.09**
Sense of danger	− 0.22***	− 0.07*	0.08*
Threats	0.03	− 0.11***	-34***
Religiosity	− 0.12***	0.08*	0.02
Education	0.10***	0.04	0.05
Gender	0.04	0.01	− 0.09***
% variability	19%	25%	27%

***p <.001; IR = individual resilience, CR = community resilience, SR = Societal resilience, WB = well-being

## Results

As a first step, we computed a Pearson correlation matrix of the examined psychological variables (Table 3). The results indicated the following: (a) The three types of resilience correlated significantly positively with each other. (b) The three types of resilience correlated significantly and positively with supporting coping indicators and significantly and negatively with suppression coping indexes. (c) The three supporting coping indicators correlated significantly and negatively with the three suppressing coping indicators. These results were the expected results.

**Table 3:** Pearson correlations among psychological variables in the Czech Republic

		CR	SR	Hope	WB	Morale	Distress	Danger	Threats
Resilience	IR	0.315***	0.155***	0.188***	0.276***	0.302***	− 0.255***	− 0.302***	− 0.147***
	CR	--	0.416***	0.355***	0.315***	0.327***	− 0.225***	− 0.223***	− 0.278***
	SR		--	0.361***	0.148***	0.378***	− 0.247***	− 0.152**	− 0.416***
Supporting coping	Hope			--	0.177***	0.268***	− 0.156***	− 0.216***	− 0.226***
	WB				--	0.342***	− 0.525***	− 0.139***	− 0.170***
	Morale					--	− 0.464***	− 0.413***	− 0.438***
Suppressing coping	Distress						--	0.227***	0.363***
	Danger							--	0.422***

***p <.001; ^a b c^ Scheffe post hoc test, ^1^ hope was not measured in Israel.

Second, we compared the level of the examined psychological variables in the Czech Republic with those variables in Slovakia (three weeks later, in November 2022) and in Israel (approximately one year earlier, during Operation Guardian the Walls, June 2021) to obtain a proportion of the level of resilience and coping indicators in the Czech Republic (Table 4).

**Table 4:** Analysis of variance among three countries regarding resilience and coping indicators

Country		IR	CR	SR	Hope^1^	WB	Morale	Distress	Sense of danger	Threats
Israel (*N* = 647)	M	3.56^a^	3.29^a^	3.89^a^	--	4.41^a^	3.33^a^	2.23^b^	2.45^b^	2.80^c^
	*SD*	*0.88*	*0.93*	*0.89*	*--*	*0.93*	*0.98*	*0.91*	*0.91*	*0.83*
Slovakia (*N* = 1011)	M	3.22^b^	2.91^c^	2.96^c^	2.49	4.26^b^	2.59^b^	2.50^a^	3.13^a^	3.32^b^
	*SD*	*0.90*	*0.70*	*1.08*	*1.02*	*0.99*	*0.95*	*0.96*	*1.06*	*0.88*
Czech (*N* = 1008)	M	3.25	3.02^b^	3.37^b^	2.39	4.42^a^	2.67^b^	2.32^b^	3.12^a^	3.08^a^
	*SD*	*0.88*	*0.71*	*1.07*	*1.03*	*0.94*	*0.91*	*0.91*	*0.96*	*0.88*
F		33.56^b^	49.47	157.34	5.50	8.87	136.65	17.65	118.63	72.81
E^2^ (Effect size)		0.025	0.036	0.106	0.003	0.007	0.093	0.014	0.026	0.052
P<		0.001	0.001	0.001	019	0.001	0.001	0.001	0.001	0.001

The results indicated the following: (a) Israelis significantly reported the highest levels of IR, CR, and SR compared with the Czech Republic and Slovakia (small effect size). (b) Slovak respondents reported a significantly higher level of hope than Czech respondents (small effect size). (c) Israelis significantly reported the highest level of hope and morale compared with the Czech Republic and Slovakia (small effect size). (d) Israelis and the Czech Republic reported a significantly lower level of distress symptoms compared with Slovak respondents (small effect size). (e) Israelis reported a significantly lower level of sense of danger compared with the Czech and Slovak respondents. (f) Slovak respondents significantly reported the highest level of perceived threats, followed by the Czech, who reported significantly higher perceived threats, compared with the Israelis.

Third, we used three sets of path analyses to examine the prediction of the three types of resilience, controlling for each other by (a) five coping indicators, (b) five demographic characteristics, and (c) both coping indicators and significant demographic characteristics (Table 2).

The results regarding coping indicators indicated the following: (a) The best predictor of IR was the sense of danger: the higher the sense of danger, the lower the IR reported, and vice versa. In addition, the higher the hope and well-being and the lower the distress symptoms reported, the higher the IR reported. The perceived threat did not significantly predict IR. Overall, the five predictors explained 16% of IR variability. (b) The best predictor of CR was the level of morale, followed by hope: the higher the levels of morale, hope, and well-being, the higher the CR reported. Distress symptoms did not significantly predict CR. Overall, the five predictors explained 22% of the CR variability. (c) The best predictor of SR is perceived threats: the higher the perceived threats, the lower the SR reported. The second-best predictor was the level of hope: the higher the hope, the higher the SR reported. Additionally, higher distress symptoms and a sense of danger were associated with lower SRs. Well-being did not significantly predict SR. Overall, the five predictors explained 15% of IR, 22% of CR, and 26% of SR variability.

Next, we used second path analysis to examine whether demographic characteristics significantly predicted resilience (Table 2). The results indicate the following: (a) The most significant predictors of IR were family income and age, followed by religiosity: the higher the income, the older the person, and the lower the level of religiosity, the higher the IR reported. (b) The best predictor of CR was family income followed by the level of religiosity: the higher income and lower religiosity, the higher CR reported. (c) The best predictor of SR was family income: the higher the income, the higher the SR reported. (d) The five demographic characteristics explain the low variability percentage of the three types of resilience: 8% of IR, 3% of CR, and 2% of CR.

Finally, we used a third path analysis to examine which of the coping indicators and the demographic characteristics together (controlling for each other) will significantly predict each of the three types of resilience (Table 2). The results indicated the following: (a) Age and family income did not predict any of the three types of resilience and were removed from the analysis. (b) Hope and sense of danger significantly predicted the three types of resilience: the higher the level of hope and sense of danger were, the higher the IR, CR, and SR reported. (c) Distress symptoms significantly predicted IR and SR (but not CR): the higher the level of distress symptoms, the lower the IR and SR reported. (d) The sense of danger significantly and negatively predicted IR and CR: the higher the sense of danger was, the lower the IR and CR reported. However, the higher the sense of danger, the higher the SR reported. (e) Perceived threats significantly and negatively predicted CR and SR (but not IR): the more perceived threats there were, the lower the CR and SR reported. (f) Religiosity significantly and negatively predicted IR: the lower the level of religiosity is, the higher the IR reported, but the higher the level of religiosity is, the higher the CR reported. (g) Education significantly and positively predicted IR: the higher the level of education, the higher the IR reported. (h) Females reported a significantly higher level of SR than males. (j) The nine variables explained 19% of IR, 25% of CR, and 27% of SR variability.

## Discussion

The current study examined the negative effects on the adult population of the Czech Republic and compared them to the negative effects in Slovakia, approximately six months after the Russian invasion of Ukraine. We also compare these negative effects with those of Israel which is often involved in combat situations. This comparison aims to assess the associations between resilience and coping indices and a war situation in geographical proximity and uncertainty regarding the future in the Czech Republic, compared with Slovakia and Israel. We examined three levels of resilience, three support, and three suppression coping indicators, as well as the contribution of demographic characteristics. The results of this study supported earlier studies showing the negative effects of war: a lower level of individual, community, and societal resilience, as well as a lower level of supporting coping resilience factors and a higher level of suppressing resilience coping factors [14].

The comparison between the Czech Republic’s sample with the Slovakian sample, which was once part of Czechoslovakia, offers a picture according to which the effect of the war in Ukraine concerns all the citizens in the countries of the region: There is a general fear of future developments. It can be assumed that Russia’s invasion of Ukraine presents a grim picture of a power headed by Putin that can decide to invade a neighboring country without being afraid of the world’s reaction. Our research indicates similar responses in Slovakia and the Czech Republic regarding resilience and coping indicators [46]. Moreover, our results suggested that perceived threat, as well as a sense of danger, were good predictors of CR and SR in the Czech Republic and Slovakia. One way to explain the importance of these two variables in the Czech Republic and Slovakia as predictors of CR and SR is to argue that these results explain the importance of not knowing about the future as important contributors to public resilience (referring to both types of resilience). Further research is required to support this explanation.

The comparison between the Czech Republic and Israel during a military conflict with Gaza raises the question of what might explain the higher resilience in Israel, as well as lower coping abilities. One way to explain these results is the claim that the citizens of Israel are used to rounds of fighting with the Palestinians, and they have developed over the years a good ability to deal with conditions of constant threats and the sense that they can trust their security forces as well as government institutions [17]. An additional way to explain the lower level of resilience and coping skills is to take into account the past of the Czech Republic [47]: The Czech Republic has a traumatic history of unfulfilled aspirations for independence in three cases– the dominance of Czech lands during the longstanding reign and oppression by the Austrian-Hungary Monarchy, the German annexation during the Second World War and the Soviet invasion in 1968 followed by tens of years of political dominance and economic drainage [48].

Our results also indicated that hope is a significant predictor of IR, CR, and SR. One should remember that hope is an expectation that the situation will improve in the future, even if in the present the individual faces difficulties and dangers [49]). People who expect a brighter future show higher support for their societal resilience [29]. Hope has the power to orient society toward the future and use resources in an effective way regarding the aspired positive goals. Establishing hope for the future is culturally not an easy task in the Czech Republic. Our research shows, however, that this uneasy task is necessary if society wants to avoid sheer survival in a defeatist powerless position that in the end produces a ruthless attitude toward other nations in need and instead develops a resilient, adaptive, and flourishing agency. However, further studies are needed to support our explanation of the contribution of history and the past events associated with the present situation.

Finally, two main results corroborate earlier studies: (a) the findings dealing with the correlations between resilience and support, as well as suppressing coping indicators, are consistent with previous studies done in different cultures as well as concerning different threats [17]. (b) The results regarding the higher prediction of resilience by psychological factors compared with demographic characteristics. One gets the impression that these results are universal. However, further research is needed to confirm these findings.

### Limitations of the study

This study, like any study, has several limitations. First, the study is a correlational-based study, which prevents researchers from concluding causality. Second, the study is based on an internet panel sample, and we have no guarantee that this sample is representative of the adult Czech Republic population. Third, the research tool is a self-report questionnaire, which may cause biases in the answers of the respondents.

## Conclusions

The first conclusion that emerges from this study concerns the negative impact of the war in Ukraine on geographically and culturally close two countries: the Czech Republic and Slovakia. This comparison reinforces our claim regarding the negative effects of the war. Although we do not have a measurement of the variables tested before the outbreak of the war, there are quite a few findings that allow us to assess, with the necessary caution, that the Ukrainian War hurts the population and is a threat to extensive aspects of the population’s life. The second conclusion points to the differences between different cultures as well as the reactions to different threats as the comparison between the Czech Republic and Israel might indicate. The third conclusion is that the associations between the resilience indices, the supporting indicators, and the suppression coping indicators are similar across cultures.

## Data Availability

The data is stored in an open-access repository and can be accessed through the following links, following registration to the website (open upon registration).Kimhi, Shaul; Koubová, Alice, 2023, “The war in Ukraine and its consequences 2022 - Slovakia” [dataset] [online], https://doi.org/…QPT, CSDA, First Version.Kimhi, Shaul; Koubová, Alice, 2023, “The war in Ukraine and its consequences 2022 - Czech Republic” [dataset] [online],https://doi.org/…XNY, CSDA, First Version.
